# A Simple Iterative Model Accurately Captures Complex Trapline Formation by Bumblebees Across Spatial Scales and Flower Arrangements

**DOI:** 10.1371/journal.pcbi.1002938

**Published:** 2013-03-07

**Authors:** Andrew M. Reynolds, Mathieu Lihoreau, Lars Chittka

**Affiliations:** 1 Rothamsted Research, Harpenden, Hertfordshire, United Kingdom; 2 School of Biological Sciences and the Charles Perkins Centre, The University of Sydney, Sydney, New South Wales, Australia; 3 Psychology Division, School of Biological and Chemical Sciences, Queen Mary University of London, London, United Kingdom; Northeastern University, United States of America

## Abstract

Pollinating bees develop foraging circuits (traplines) to visit multiple flowers in a manner that minimizes overall travel distance, a task analogous to the travelling salesman problem. We report on an in-depth exploration of an iterative improvement heuristic model of bumblebee traplining previously found to accurately replicate the establishment of stable routes by bees between flowers distributed over several hectares. The critical test for a model is its predictive power for empirical data for which the model has not been specifically developed, and here the model is shown to be consistent with observations from different research groups made at several spatial scales and using multiple configurations of flowers. We refine the model to account for the spatial search strategy of bees exploring their environment, and test several previously unexplored predictions. We find that the model predicts accurately 1) the increasing propensity of bees to optimize their foraging routes with increasing spatial scale; 2) that bees cannot establish stable optimal traplines for all spatial configurations of rewarding flowers; 3) the observed trade-off between travel distance and prioritization of high-reward sites (with a slight modification of the model); 4) the temporal pattern with which bees acquire approximate solutions to travelling salesman-like problems over several dozen foraging bouts; 5) the instability of visitation schedules in some spatial configurations of flowers; 6) the observation that in some flower arrays, bees' visitation schedules are highly individually different; 7) the searching behaviour that leads to efficient location of flowers and routes between them. Our model constitutes a robust theoretical platform to generate novel hypotheses and refine our understanding about how small-brained insects develop a representation of space and use it to navigate in complex and dynamic environments.

## Author Summary

Pollinating bees, along with bats, hummingbirds, rodents and primates, typically develop circuits (traplines) to visit multiple foraging sites in an efficient stable sequence. The question of how animals encode and process spatial information to develop these impressive foraging patterns remains poorly understood. Previously we showed that an iterative improvement heuristic model of bumblebee traplining can replicate the establishment of stable routes by bees between flowers distributed over several hectares. Here we tested the model against a variety of datasets with different configurations of flowers and found it to give good agreements with all these observations. We have thus shown how these complex dynamic routing problems can be solved by small-brained bees using simple learning heuristics and without acquiring a ‘map-like’ memory. The proposed heuristic shows how bees develop optimal routes simply by following multi-segment journeys composed of learnt flight routines (local vectors), each pointing towards target locations (flowers) and coupled to a visual context (landmarks or panoramas). Such a decentralized representation of space relying on learnt sensorimotor routines is akin to ‘route-based’ navigation as described in desert ants, where spatial information is thought to be processed by separate, potentially modular, guidance systems.

## Introduction

Bees, bats, hummingbirds, rodents and primates which exploit patchily distributed foods that replenish over time often visit resource locations in predictable sequences [1]–[12]. In pollinating insects, such as bumblebees, these traplines are often the shortest circuits to visit all the known flower locations exactly once before returning to the nest and so are solutions of the well-known travelling salesman problem (TSP) [13]. Just how these animals solve this problem with relatively low computational power has long been a mystery [14]–[16]. The TSP is, after all, one of the most intensively studied problems in combinatorial optimization [13]. There are no efficient algorithms for even solving the problem approximately (within a guaranteed constant factor from the optimum) because the problem is NP-complete (nondeterministic polynomial time complete) and it is believed that there is no algorithm that can find a solution where the processing time increases as a finite order polynomial in N [17]. The most direct approach would be to try all of the permutations and then select the shortest one, but this becomes impractical even for only 20 locations as the number of permutations is 20!. Nonetheless, approximate solutions can be found using linear programming methods, neural networks, simulated annealing and genetic algorithms [17]. The best approximate algorithms can typically find solutions within 1–2% of the optimum but these are unlikely to be implemented by biological organisms because they are computationally demanding [13].

Several algorithms have been proposed to explain how animals might optimise multi-location routes [18]. Perhaps the simplest candidate model of bumblebee trapline development is the ‘nearest neighbour’ or ‘greedy’ heuristic, in which a model bee chooses the nearest unvisited flower as its next move until all flowers have been visited. It has been suggested that this simple heuristic explains the routing behaviour of some animals [14], [19]–[21] but it is incompatible with observations of bumblebees foraging at various spatial scales [15], [16]. No better is a simple random ‘*k*-opt’ iterative improvement heuristic [22] in which a model bee (1) tries to improve the route between known flowers by randomly shuffling the order in which a number (k) of randomly selected flowers are visited, and (2) the route change is kept if the new route is shorter than the previous one (otherwise it is rejected). This heuristic significantly over-predicts the number of foraging bouts executed before the first appearance of an optimal (shortest-path length) foraging route and unlike bumblebees does not create stable traplines [16]. Recently we proposed that bumblebees use a simple learning heuristic (‘The Basic Traplining Heuristic Model’) to develop optimal traplines between distant feeding locations in the field. This heuristic is based on our general knowledge of bee navigational strategies [23], including bees' tendency to discover flowers in relation to their distance to the nest [16], the fact that they learn sequences of vector flights between familiar locations using the visual context (landmarks and/or panoramas) [24]–[26], and their ability to measure travel distances through the image movement over their retina (optic flow) experienced during flight [27], [28]. In this heuristic, model bees try a limited number of possible route iterations, so that route segments (between pairs of flowers) that shorten the overall route are reinforced in memory, while others are abandoned, allowing bees to develop an adaptive (and occasionally optimal) ‘trapline’ whilst retaining some ability to adjust their route in response to changes in the spatial configuration of flowers [16]. This model predicts that bees: (1) occasionally visit fewer than all flowers especially during early bouts; (2) regularly revisit empty flowers during the same bout; (3) decrease their frequency of returns to just-visited, empty flowers with experience; (4) establish stable optimal routes in some spatial configurations but not others; (5) can sequentially adjust their routes to incorporate newly discovered flowers in an optimal way when the number of locations is relatively small. Quantitative evaluation of the simulated data with bees' optimisation performances at an array of five artificial flowers arranged in a regular pentagon (50 m side length) set up in the field showed full agreement (as quantified by p-values for the probability of the data given the model) for the number of bouts: (1) to the first appearance of an optimal sequence; (2) the number of bouts to the stabilization of an optimal sequence into a trapline; (3) the number of different routes experienced; (4) the net travel length per bout; (5) the number of revisits per bout: and (6) the similarity indices between successive bouts [16]. In accordance with empirical data [16] the model also predicts correctly that bee flight paths are constrained by previous experience and that bees cannot compute entirely novel solutions quickly. Our simple model relies on reactive navigation rules rather than a “cognitive map” and might require relatively low cognitive demands. It may therefore provide an important indication of how bumblebees encode and use spatial information when developing traplines. Nonetheless, detailed analyses of the model are necessary to refine our understanding of this strategy and clarify whether similar learning heuristics apply to bumblebees foraging at different spatial scales and configurations.

**Figure 1:**
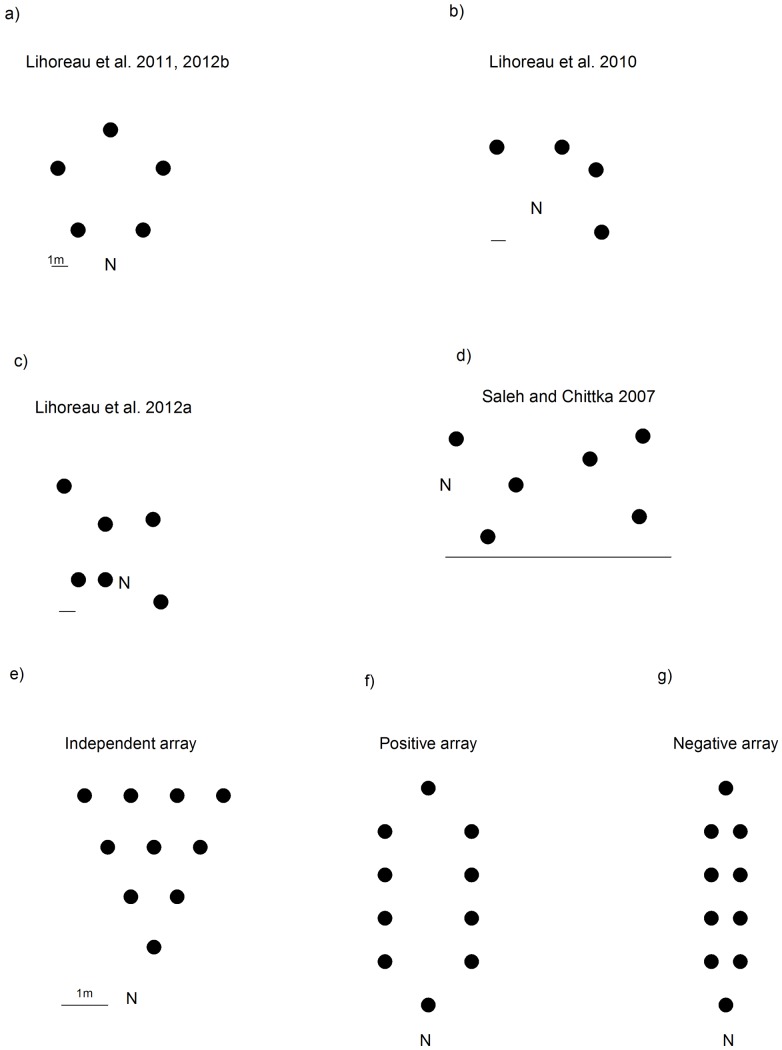
Schematics of the artificial flower arrays used in the experimental studies under investigation. **a–c**) Lihoreau et al. [15], [16], [31], [32], **d**) Saleh and Chittka [29] (upper panels) and the **e**) ‘independent’, **f**) ‘positive’ and **g**) ‘negative’ arrays of flower (•) used in the study of Ohashi et al. [14] (lower panel). The position of the nests (N) is indicated. 1 m scale bars are shown. In the ‘independent’ array the flowers were arranged so that bees can choose distance and turning independently; in the ‘positive’ flower array, proximity and directionality were positively linked, so that the nearest neighbouring flower could be reached by straight-ahead movement; and in the ‘negative’ flower array, proximity and directionality were negatively linked, so that choosing the nearest neighbour flower as the next flower to visit required bees to make turns.

In this paper we show that our model is consistent with all published observations [14]–[16], [29]–[32] made at small spatial scales that have established how bumblebees optimize their routes between fixed resources [15], [29], re-optimize their routes after identifying a new resource [31] and how and when bees prioritize high-reward resources [32] in various floral arrays (Fig. 1). These studies have been performed in flight rooms where bees could potentially see all artificial flowers from any vantage point. The dimension of these flight rooms varied. The study of Saleh and Chittka [29] was carried out in an indoor flight arena measuring 105 (L)×75 (W)×30 (H) cm. The studies of Ohashi et al. [14] were carried out in an indoor flight cage (788×330×200 cm). The studies of Lihoreau et al. [15], [16], [31], [32] were carried out in a greenhouse (870×730×200 cm). We begin by showing that the model can account for the observed increasing propensity of bees to find optimal routes with increasing spatial scale and show that is predicts correctly the formation of stable optimal traplines for some arrangements of flowers but not others [16], [32]. We then show that our model predicts that bumblebee flight patterns made during the course of a day (between 65 and 80 foraging bouts) between 10 or fewer irregularly distributed flowers will often converge onto the shortest possible path or find good approximate solutions of it. This is an impressive feat because there are 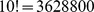 different ways of travelling between 10 flowers. We also show that the model predicts that after locating more flowers than necessary to fill their crop capacity (nectar stomach size), bumblebees can develop highly effective traplines, by visiting only a set of flowers with an appropriate spatial configuration. Finally, we show that the bee searching behaviour is consistent with their adopting an optimal searching strategy.

## Methods

### The basic traplining heuristic model

The basic model – an iterative improvement heuristic - is described in Lihoreau et al. [16]. The heuristic mimics the behaviour of a bumblebee collecting nectar in a stable array of flowers and returns to its nest over multiple consecutive bouts. At the end of each foraging bout, flowers replenish with a new load of nectar. At each stage, a model bee chooses to move between flowers according to six assumptions: (1) the bee can uniquely identify each flower using information from path integration and/or the visual context (landmarks, panoramas) [24], [25]; (2) the bee has a finite probability of using transition vectors joining each pair of flowers; (3) the initial probability of using a vector depends on the distance between the two flowers (in our simulations these probabilities are inversely proportional to the squared distance between flowers and are normalized with respect to all flowers); (4) the bee computes the net length of the route travelled using optic flow (odometer) [27], [28], by summing the distances of all vectors comprising the flower visit sequence; (5) having completed a route passing through all the flowers at least once (and thus filled their crop capacity), the bee compares the net length of the current route to the net length of the shortest route experienced so far that passes through all the flowers; (6) if the new route is no longer, the probabilities of using the vectors forming this new route in the next foraging bout are multiplied by a common factor and then all probabilities are rescaled with respect to all flowers so that they sum to unity. Repeating the shortest route therefore reinforces it.

### Quantifying model agreement with observations: p-values

The model was used to predict the distributions of the number of bouts before the first appearance of an optimal (shortest) route (if found) and the number of bouts before the optimal routes became established as the only foraging route stabilised. These distributions were based on 1000 runs of the model. These distributions were then used to calculate the probability of a real bee doing at least as well given the model (i.e. the null hypothesis) is correct, i.e., the numbers of bouts/routes were ordered and then the ranking of the real bee observation was determined. This probability is a p-value. A p-value of 0.3 means that the numbers of bouts/routes for the real bee was equal to the 70^th^ % quickest result in the numerical simulations. The model can be rejected if the p-value is lower than 0.05. Typically p-values are much larger than this threshold.

### Asymmetry index

Aside from comparisons with our own data [15], [16], [31], [32] we will also compare our model with the empirical study of Ohashi et al. [18]. For each pair of flowers (*i*, *j*), Ohashi et al. [18] recorded the numbers of transitions from flower *i* to flower *j*, and from flower *j* to flower *i* made during 9–10 successive foraging trips. These transition matrices were characterised by asymmetry indices 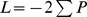, where *P* is the binomial probability of the observed departure from a 1∶1 expectation of the observed number of transitions, i.e. if there were *N* transitions between flowers *i* and *j* with *n* transitions being from flower *i* to flower *j* then the associated binomial probability
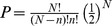
. *P* values with fewer than 6 observations were omitted. The asymmetry indices were then standardized by dividing by the number of pairs tested, which varied with foraging stage and among the bees. This standardization was not mentioned in the paper by Ohashi et al. [14] (K. Ohashi, private communication).

## Results

### Motivation and spatial scale

Lihoreau et al. [32] reported on bee optimisation performances in an array of five artificial flowers arranged in a regular pentagon (5 m side length) in a flight room (Fig. 1a). All flowers had the same reward value and their spatial arrangement was similar to the one used in the field study of Lihoreau et al. [16]. However, unlike at the field scale, at these scales the bees could potentially detect all of the flowers visually from any location. Nonetheless, stable optimal routes (visiting each flower once and returning to the nest using the shortest possible path) only became established after bees had made 34 or more foraging bouts. This is significantly more than in the field experiment using a scaled-up arrangement of flowers with side length 50 m where around 26 bouts were required for the establishment of the optimal route [16]. This suggests that a bee's “motivation” to optimise its route increases with spatial scale because the costs of travelling suboptimal routes are lower when flying a few metres than when flying several hundred metres [16]. We tested whether this difference in tendency to optimise can be captured by the model by adjusting the common factor by which vectors are reinforced each time a short route is found (see Methods).

Good model agreement with the data collected at the field-scale was only obtained when the probability enhancement factor fell between about 1.5 and 4 [16]. Smaller probability enhancement factors, less than about 1.1, brought the model into good agreement with the data collected in the flight room by Lihoreau et al. [32] (Table 1). This suggests that the probability enhancement factor in our model is scale-dependent and can be associated with motivation to optimize a route. Similarly, good model agreement with the data collected in the same flight room with 4 flowers [31] and with 6 flowers that were between 1 and 10 m apart [32] and with data collected in a smaller flight arena with 6 flowers less than 1.0 m apart [29] was only obtained with probability enhancement factors less than 1.5 (flower arrangements shown in Fig. 1b–d).

**Table 1:** Comparisons between empirical and simulation data for three traplining characteristics at a pentagonal arrangement of flowers (Fig. 1a).

Diagnostic	Probability of the data given the model with an enhancement factor = 2	Probability of the data given the model with an enhancement factor = 1.1
**No. bouts until first appearance of an optimal route**	0.32–0.85	0.20–0.76
**No. of bouts until first appearance of 3 consecutive optimal routes (stability)**	0.19–0.97	0.85–1.00
**No. of distinct routes**	0.00–0.16 (Reject)	0.40–1.00

The empirical data comes from [32] (Expts. 2 & 3, bouts 1–40, 10 bees tested, Fig. 1) and comparisons are quantified by p-values, which are the probabilities of the empirical data given the model.

**Figure 2:**
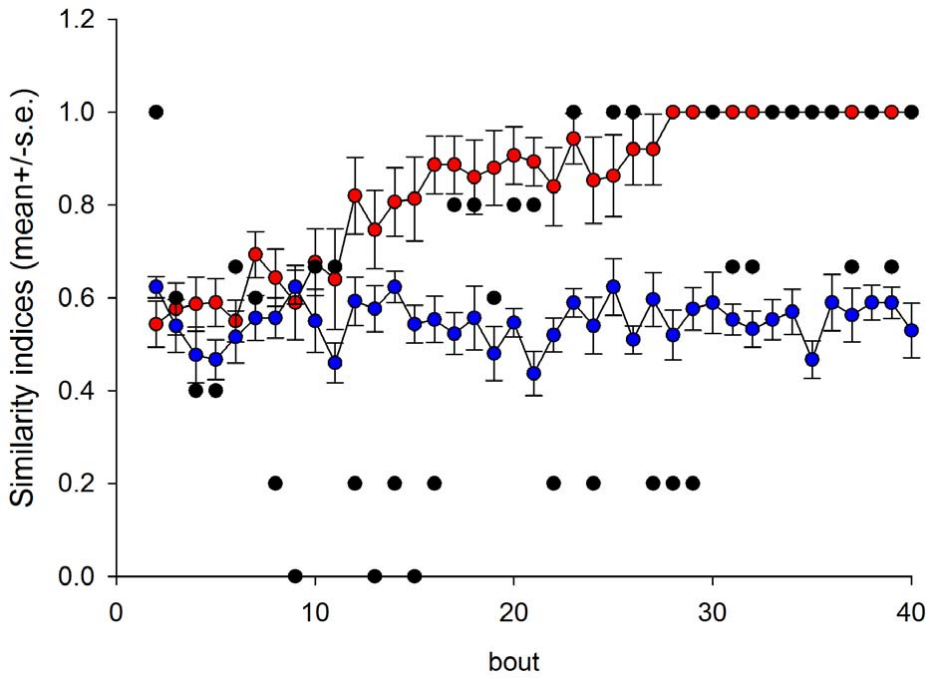
Predicted and measured similarity indices for consecutive sequences of floral positions visited by bees in a 5-flower array. The similarity index quantifies the similarity between pairs of flower visitation sequences. They take into account the length of sequences and the order of visits to flowers, and range between 0 (completely different sequences, e.g. 123 vs 456) and 1 (identical sequences, e.g. 12345 vs 12345). Predictions are shown for probability enhancement factors of 2 (red circles) and 1.1 (blue circles) and for an individual real bee [32] (black circles). The model with an enhancement factor of 1.1 does not lead to gradual increases of route similarity as observed in the real bee. The model with a probability enhancement factor of 2 is seen to capture well the overall dynamics of how a bee's tendency to repeat visitation sequences increases with experience. However, the model does not fully capture the bees' occasional tendency to depart completely from established routes and explore entirely dissimilar routes in this experiment.

We then examined how a bee's tendency to repeat visitation sequences increases with experience using a similarity index (SI), described in Saleh and Chittka [29], which quantifies the similarity between pairs of flower visitation sequences. SI takes into account the length of sequences and the order of visits to flowers. SI ranges between 0 (completely different sequences, e.g. 123 vs 456) and 1 (identical sequences, e.g. 12345 vs 12345). The model was in closest agreement with the observational data [29], [32] (i.e. the p-values were largest) when the probability enhancement factor was about 1.1 (Fig. 2). For example, for the case of Lihoreau et al. (Expt. 2, Bouts 1–40) [32], p-values ranged between 0.16 and 0.43 when the probability enhancement factor was 2.0 and ranged between 0.43 and 0.62 when the probability enhancement factor was 1.1

### Stable optimal traplines cannot be established for all spatial configurations of flowers

Ohashi et al. [14] reported on the ontogeny of foraging paths in 3 different spatial configurations of 10 flowers that were less than 1.0 m apart (Fig. 1e–g). In their ‘independent’ array, 10 flowers were arranged in a triangular pattern so that bees can choose distance and turning independently; in the ‘positive’ flower array, proximity and directionality were positively linked, so that the nearest neighbouring flower could be reached by straight-ahead movement; and in the ‘negative’ flower array, proximity and directionality were negatively linked, so that choosing the nearest neighbour flower as the next flower to visit required bees to make turns. Ohashi et al. [14] reported that bumblebees preferred to choose short distances over straight flights and showed little plasticity in this regard, and as a consequence are less able to approximate the TSP solution in a ‘negative’ flower array compared to other arrays. Ohashi et al. [14] reported that 3 out of the 6 bees tested in the positive array established optimal traplines, just 1 out of 5 bees tested in the independent array established an optimal trapline and none of 5 bees tested in the negative array established optimal traplines. Our model is consistent with these observations. After 65 bouts, the model predicts that about 80% of the bees will have established stable optimal traplines in the positive array; 10% of the bees will have established stable optimal traplines in the independent array; and no (0 out of 100) bees will have established stable optimal traplines in the negative array. The latter prediction arises because the initial probability of using a vector depends on the distance between the two flowers (in our simulations these probabilities are inversely proportional to the squared distance between flowers). In accordance with the observations of Ohashi et al. [14] our model predicts that stable optimal traplines cannot be established for all spatial configurations of rewarding flowers. This is true at the scale of the experiments (probability enhancement factor 1.1) and at the field scale (probability enhancement factor 1.5).

Ohashi et al. [14] reported that the asymmetry index increased with foraging experienced and for this reason they reported on median rather than mean values of the standardized asymmetry index. The median standardized asymmetry indices for the positive, independent and negative arrays were 5.47±1.10 (mean±s.e.), 4.37±0.37 and 4.56±0.56. Comparable model predictions (with overlapping ranges) are obtained when the probability enhancement factor is less than about 1.5. Model predictions for a probability enhancement factor of 1.5 are 5.65±2.69, 4.86±1.78 and 5.58±2.22.

### Finding solutions to the Travelling Salesman Problem

**Figure 3:**
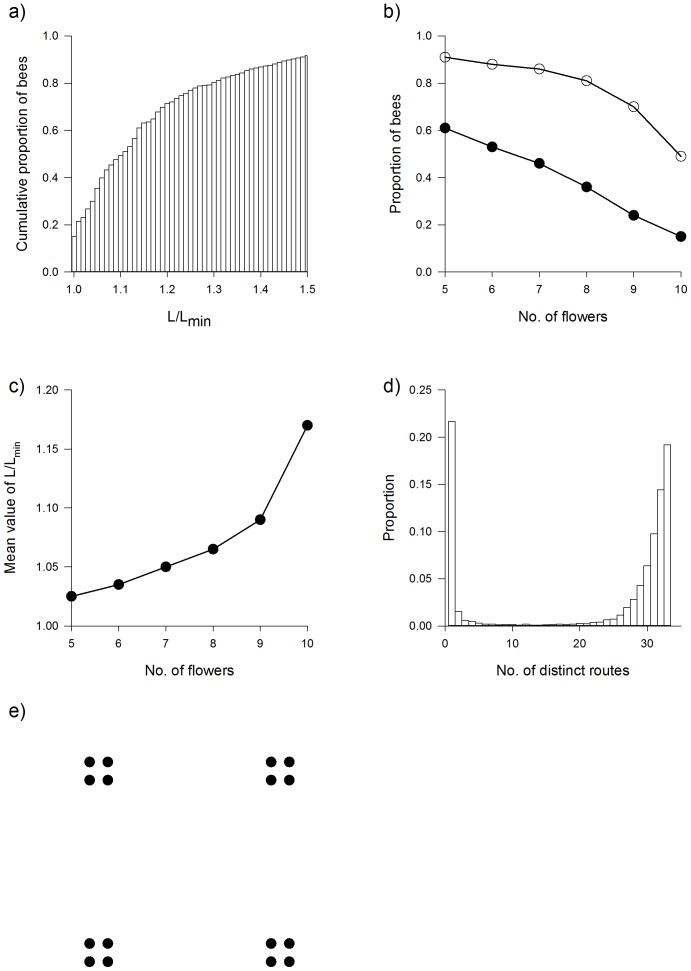
Predicted flight performance in the presence of 10 flowers that are randomly and uniformly distributed within a square patch. **a**) Cumulative proportion of model bees with traplines of length L/L_min_ is the length of the shortest possible routes between the flowers. **b**) Predicted optimisation performance of bees in relation to the number of flowers the bees must visit to fill their crop. Proportion of model bees that find solutions to the travelling salesman problem (•) and find good approximate solutions (i.e. have flight lengths that are no more than 10% longer than the shortest possible flights) (○). **c**) The average value L/Lmin in relation to the number of flowers. **d**) Distribution of the numbers of distinct routes taken during bouts 33–65 between 10 flowers. **e**) Schematic of flower array used to evaluate predicted optimization performance of bees foraging for patchily distributed resources.

Having demonstrated good agreement between our model and various datasets of the literature, we then used the model to predict the optimization performance of bees when foraging on randomly rather than regularly distributed flowers. The simulation data were obtained for 100 different random arrangements of N flowers, and for 100 bees per arrangement. The probability enhancement factor is two, as this brought the model into good agreement with the observations. The model predicts that the numbers of bees that find the optimal path between N randomly distributed flowers during the course of a day (65 foraging bouts) decreases as the number of flowers increases but remains sufficient even for 10 flowers (Fig. 3a,b). Nonetheless, some of the random arrangements of flowers form ‘negative’ arrays as proximity and directionality were negatively linked and in these cases no model bees found an optimal route. For other random arrangements of the flowers, almost all of the models bees found the optimal route. The average path length as a proportion of the minimum path length increases as the number of flowers increases but is less than 1.25, the value obtained using the nearest neighbour algorithm, when there are 10 or fewer flowers (Fig. 3c).

It is computationally prohibitive to test the optimality of routes made between 20 or more flowers. Nonetheless, most (about 98.5%) of the shortest routes made between 20 randomly distributed flowers that were found by the model bees during the course of a day (65 foraging bouts) could be shortened by simply switching the order in which two of the flowers were visited, i.e. by changing the flower visitation sequence 5875431… to 5835471… by switching the order in which flowers 3 and 7 are visited. The routes that could not be shortened in this way could be the shortest of 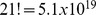 possible routes between the flowers. After 2 days of foraging (130 foraging bouts) without overnight memory loss, about 96.5% of the shortest found routes could be shortened by such pairwise switching of the visitation sequences. Model bees that do not find optimal traplines gradually reduce the number of distinct routes taken between the flowers, but generally do not form stable non-optimal traplines during the course of a day (Fig. 3d).

Bees foraging on several distant patches are predicted to eventually minimize overall travel distances between patches but not necessarily travel distances within patches. Most model bees (with constant probability enhancement factors) foraging on 4 patches located at the corners of a square (Fig. 3e) did, for example, follow optimal clockwise or anticlockwise routes when flying between patches during the course of the day. Optimal clockwise or anticlockwise routes were flown about 70% less frequently within patches.

### Developing efficient traplines by selecting a set of flowers with an appropriate spatial configuration

**Figure 4:**
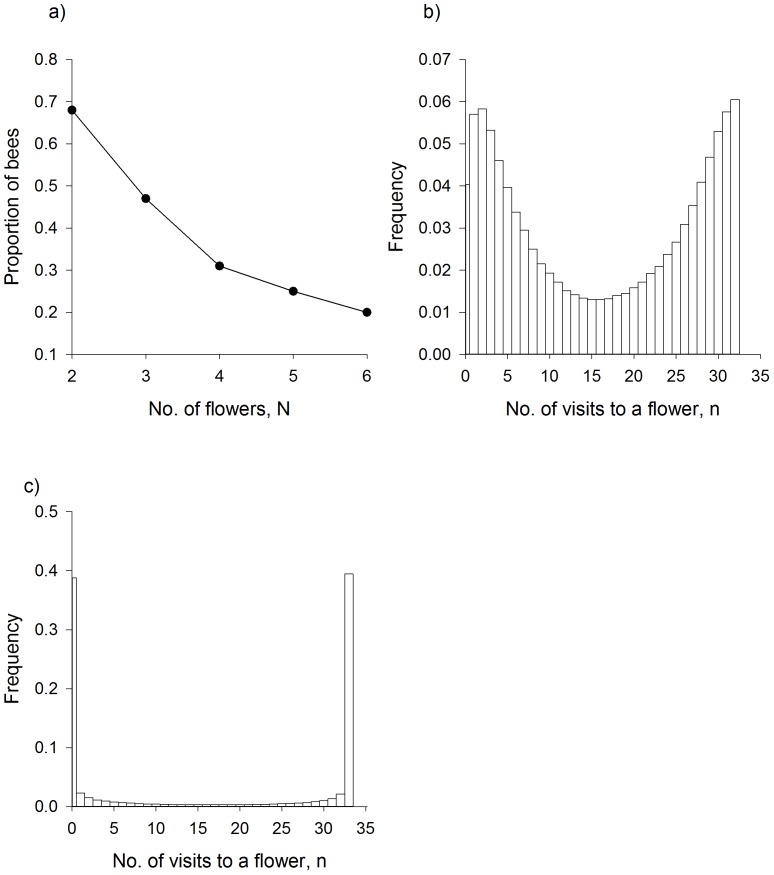
Predicted flight performance in the presence of 8 known flowers that are randomly distributed within a square patch. **a**) Predicted optimisation performance of bees in relation to the numbers of flowers, N, the bees must visit to fill their crop The figure shows the proportion of model bees that find the shortest possible routes between N flowers and the nest during the course of a day (65 foraging bouts). **b, c**) Predicted frequency of occurrence of making n visits to a flower during bouts 1–35 and bouts 35–65 when a bee must visit 4 flowers to fill their crop.

The aforementioned results together with the observations of Ohashi et al. [14] (3 different arrangements of 10 flowers in a small flight cage) suggest that a bee's ability to optimise its foraging route may depend largely on how it selects a set of flowers or patches of flowers to visit. If it has sufficient options, a bee might select a set of flowers or patches for which the route between them can be optimized. This tendency would be limited by the number of located resources and possibly because bumblebees avoid intensive overlap of their foraging areas with competitors [33], [34]. We used the model to predict the optimization performance of bees when the crop capacity is filled after visiting some but not all known flowers. When the crop capacity is filled, a model bee returns directly to the nest. The simulation data were obtained for 100 different random arrangements of the 8 flowers, and for 100 bees per arrangement. The probability enhancement factor was two. The model predicts that a significant proportion (≥20%) of bees can find the optimal route between a few known randomly distributed flowers during the course of a day (65 foraging bouts) (Fig. 4a). Irrespective of whether or not the optimal route is found, the model bees do tend to form stable traplines so that some flowers are repeatedly revisited during the day whilst others are largely neglected (Fig. 4b,c). In accordance with the observations of [29] [6 flowers in a small flight cage], the non-optimal routes are predicted to be dependent upon an individual's foraging history.

### Trade-off between travel distance and prioritization of high-reward sites

**Figure 5:**
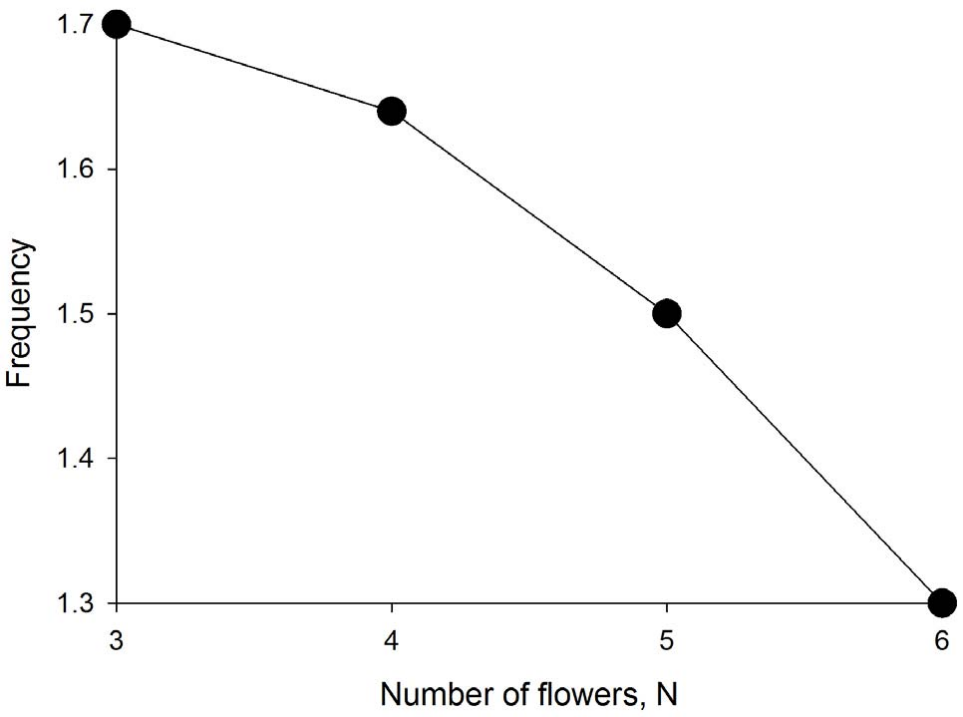
Predicted flight performance in the presence of 8 known flowers are that randomly distributed within a square patch. 2 flowers are co-located and are a proxy for single highly rewarding flower. Frequency of visiting the most rewarding flower in relation to the numbers of flowers, N, the bees must visit to fill their crop (•). The line is added to guide the eye.

Lihoreau et al. [32] demonstrated that traplining bees trade-off between minimizing travel distance and prioritization of the most rewarding locations. After the introduction of a highly rewarding flower to the pentagon array, the bees re-adjusted their routes visiting the most rewarding flower first provided that the departure distance from the shortest route was sufficiently small (18%). However, when routes optimizing the initial rate of reward were much longer (42%), bees prioritized short travel distances. This behaviour can be captured qualitatively by the model by enhancing the initial value of the probability for flying between the nest and the highly rewarding flower. If there are more flowers than necessary to fill a bee's crop capacity and if flowers vary in their reward value, then the model bees tend to establish stable optimal traplines at the field scale and the most rewarding flowers are visited more frequently than are the least rewarding flowers. The tendency to prioritise the most rewarding flowers decreases as the number of flowers necessary to fill a bee's crop capacity increases, i.e. as the typical reward value decreases (Fig. 5).

### Optimal searching behaviours

Naïve bees need to search for the flowers, and experienced bees were found to search after removal of a found artificial flower [16]. Lihoreau et al. [16] were the first to record these searching flights and this allows for the development of a model of bee searching behaviour during trapline development. These searches comprise loops centred on the location of a found flower or the location of a missing flower [16]. The size of a typical loop tends to decrease with experience (bout number) eventually becoming comparable with the ‘learnt’ typical distance between flowers. The typical size of loops made by experienced bees searching after removal of a flower also appears to be comparable with the learnt distance between flowers [16]. Here, using a simple mathematical model, we show that a looping searching strategy is near optimal for the location of flowers when the expected distance between flowers is known (has been learnt from experience), and when the typical loop size is comparable with that distance. Our finding suggests that the naïve bees gradually optimize their loop searching strategy by utilizing information they gain about the distance between flowers, and then this use optimal strategy when searching after removal of a flower, i.e., when searching after a known food source becomes depleted.

In the model of searching developed here, a bee travels out from the origin of its search (the nest initially or the location of a previously found or missing flower) along a randomly orientated straight-line (the outward leg of a loop) whose length, 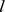, is drawn from an exponential distribution 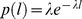 where 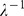 is the average length of the outward leg of a loop. The bee then flies continuously in that direction whilst constantly searching for the flower. The search ends if the flower lies within a ‘direct perception’ distance, r, of the bee. If the flower is not sighted, the bee stops after traversing the distance, 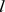, and returns to the origin of its search by retracing its outward flight. It then randomly chooses a new direction and a new distance before travelling out again. The search is centred on the origin because, initially at least, that is the most likely location of the flower.

The number of loops, 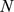, in a searching flight can be estimated by simply noting that a search will end when the length of the longest loops, 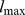, become comparable with the distance from the centre of the search to the flower, 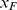, i.e. by noting that 
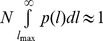
 which dictates that a loop with length longer than 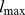 occurs at most once in the search pattern [35]. This condition gives 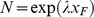. It follows from this that the average length of an entire search path, 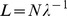, is given by 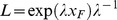 and so is minimal when 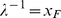, i.e. when the average length of the outward leg of a loop equals the expected distance to the flower. This optimization is not specific to exponential loop-length distributions and has been validated in numerical simulations (data not shown). It can be understood intuitively. If loops tend to be shorter than the distance between flowers then the search will be very long because most loops will fall short of the nearest flower so prolonging the search (sufficiently long loops will be rare). If loops tend to be longer than the distance between flowers then the search is unnecessarily long as the bee will frequently fly beyond where a new or missing flower is expected to be. We could not actually test this model in detail because of the small amount of empirical data available.

## Discussion

Previously we showed that a simple iterative improvement heuristic model of bumblebee traplining can accurately replicate the establishment of stable foraging routes by bees between five flowers distributed over several hectares [16]. In this paper, we have confronted this model to five other datasets from the literature and demonstrated that it also captures the development of traplines at smaller spatial scales in different arrangements of flowers. We showed that the model predicts correctly the formation of stable optimal traplines for some arrangements of flowers but not others, and accounts for the observed increasing propensity of bees to find optimal routes with increasing spatial scale [16], [32]. Bees foraging on several distant patches are therefore expected to eventually minimize overall travel distances between patches but not necessarily travel distances within patches. The model can also be modified to account for the observed trade-off between travel distance and prioritization of high-reward sites [32].

The model predicts that bees can, during the course of a day (ca. 65 foraging bouts), find solutions or good approximate solutions to the TSP. These approximate solutions tend to have a certain level of instability because bees never quite abandon interfacing exploration with the exploitation of known resources in a known order, so that an optimal route can be followed by a sub-optimal route. The bumblebee algorithm as encoded by our model also becomes impractical for 20 or more locations. However, it is effective for up to about 10 locations, which in practice could facilitate the linking up of flower patches or large plants (trees or bushes) with an optimal or near optimal routes rather than individual flowers as bumblebees typically visit 100's or even 1000's of individual flowers before returning to their nests [36]. The algorithm is less effective at linking up individual flowers within a patch. The model also predicts that after locating multiple flowers whose total nectar volume is in excess of their crop capacity, bumblebees can develop highly effective traplines, by visiting only a set of flowers with an appropriate spatial configuration. This selection arises naturally within our model without the need for additional modelling.

Despite a long history of research on bee learning and navigation, most knowledge has been deduced from the behaviour of foragers travelling between their nest and a single feeding location [23]. Only recently, studies of bumblebees foraging in arrays of artificial flowers fitted with automated tracking systems have started to describe the learning mechanisms underpinning complex route formation between multiple locations [14]–[16], [29]–[32]. The demonstration that all these observations can be accurately replicated by a single learning heuristic model holds considerable promises to further investigate these questions and fill a major gap in cognitive ecology [37]. We also provided theoretical evidence that the searching strategies employed by bumblebees and reminiscent of those seen in desert ants and in desert isopods [38], [39] become optimized over time as the bees gain knowledge about the spacing between flowers. They can be contrasted with the ‘scale-free’ strategies adopted by honeybees when searching for their hive or after the only known food becomes depleted; situations lacking a characteristic spatial scale [40], [41]. Future developments of our modelling platform will allow us to generate specific empirically testable predictions about how different organisations of spatial memory might produce different movement patterns and optimisation dynamics by bees in more ecologically relevant situations, for instance in the presence of competitors or in environments containing resources of different nutritional values. In the future, by incorporating searching behaviours and flight trajectories into the model, we will be able to make even more robust and precise predictions about trapline development.
